# The treatment of Barrett’s esophagus

**Published:** 2009

**Authors:** Petre Hoara, Cristina Gindea, Rodica Birla, Adrian Mocanu, Emmanouil Tavlas, Silviu Constantinoiu

**Affiliations:** * “Carol Davila” University of Medicine and Pharmacy, Bucharest, Romania

**Keywords:** Barrett’s esophagus, treatment, esophageal reflux

## Abstract

The reflux of the gastric juice in the esophagus can determine the injury of the esophageal epithelium. When the healing of the lesion is done by replacing the normal squamous epithelium with columnar epithelium, the entity is called Barrett’s esophagus (BE). Although controversial, some studies showed 0,5% per year the incidence of the esophageal adenocarcinoma in patients with BE, 30 times more often than general population. Taking into consideration the possible development of an adenocarcinoma, the patients with Barrett’s esophagus require endoscopic surveillance after a standardized protocol. There is still much controversy about the treatment of patients with Barrett’s esophagus, especially in the presence of dysplasia. The aims of the treatment are gastro-esophageal reflux symptoms control, healing of associated esophagitis and prevention of development of adenocarcinoma.

## Introduction

**Fig. 1:**
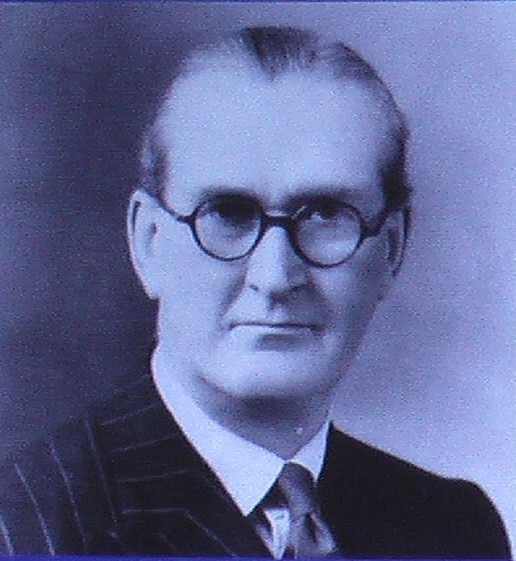
Norman Rupert Barrett (1903 - 1979)

The reflux of the gastric juice in the esophagus can determine the injury of the esophageal epithelium. When the healing of the lesion is done by replacing the normal squamous epithelium with columnar epithelium, the entity is called Barrett’s esophagus (BE), named after the British thoracic surgeon, Norman Rupert Barrett, who dedicated a great part of his activity to the study of these lesions. (**Fig. 1**).

## Endoscopy

This epithelium is an incomplete form of intestinal metaplasia, which contains several types of cells, with gastric, intestinal or colonic characteristics. Although this type of epithelium is more resistant to the gastro-esophageal reflux than squamous epithelium, metaplasic cells carry malignant potential. It is known that gastro-esophageal reflux disease and BE are the most important risk factors for the development of esophageal adenocarcinoma. Almost 10% of the patients with chronic gastro-esophageal reflux have BE. [**1**,**2**].

Although controversial, some studies showed the incidence of the esophageal adenocarcinoma in patients with BE to be 0,5%/year, 30 times more often than general population [**3**].

**Fig. 2:**
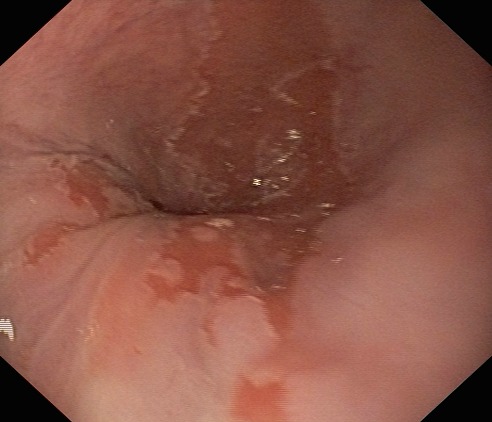
Columnar epithelium – Barrett’s esophagus

The definition of BE has suffered several changes over the years, the first definition, in 1975, being “columnar metaplasia anywhere in the esophagus”. In 2006, The British Society of Gastroenterology defined BE as: esophagus in which any portion of the normal squamous lining has been replaced by a metaplastic columnar epithelium which is visible macroscopically. In order to make a positive diagnosis of “Barrett’s esophagus”, a segment of columnar metaplasia of any length must be endoscopically visible above the esophago-gastric junction and confirmed or corroborated histologically (**Fig. 2**).

The presence of area of intestinal metaplasia, although frequent, it is not obligatory. To better understand the definition, the notion of eso-gastric junction should be defined. This is the place where the tubular esophagus meets the stomach pouch. It is of two kinds: muscular junction: manometrically defined as the distal portion of the lower esophageal sphincter (LES); mucosal junction: the squamo - columnar junction - Z line - located 1-2cm above the muscular junction. EB should be endoscopically suspected when the Z line does not coincide with the eso-gastric junction. Diagnosis of Barrett’s esophagus is endoscopically suspected and is confirmed by histopathological examination of biopsy pieces. Patients with Barrett’s esophagus may present with a single island of metaplasia to a long BE segment, of over 3cm. These lesions represent an advanced stage of gastro esophageal reflux disease. 44% of the patients have gastric hypersecretion.

**Pathology**

**Fig. 3:**
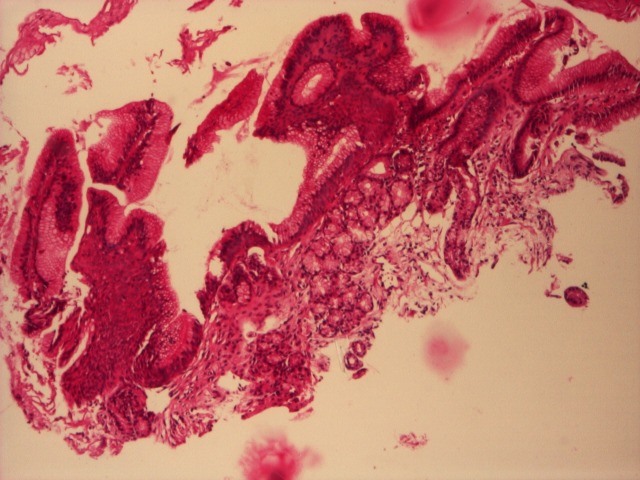
Barrett’s esophagus, negative for dysplasia, HE, 4x

**Fig. 4:**
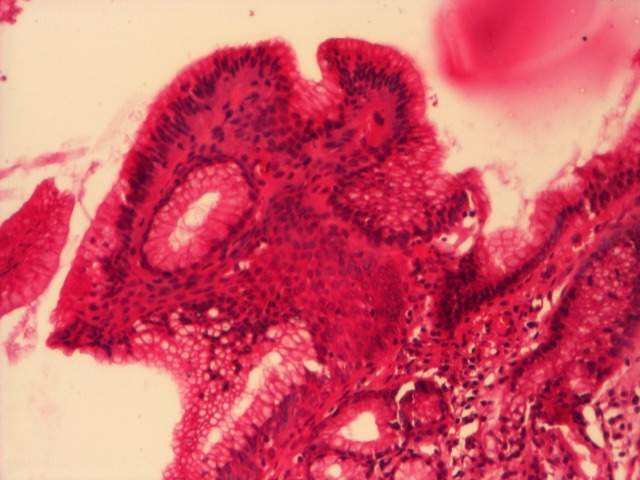
Barrett’s esophagus, low-grade dysplasia, HE, 4x.

Riedell was the first to show the presence of dysplasia in some patients with BE, and divided the patients into three categories:

- BE without dysplasia (**Fig. 3**).

- BE with undefined dysplasia

- BE with dysplasia 

- The dysplasia in patients with BE can be:

 o Low-grade dysplasia (**Fig. 4**)

o High grade dysplasia

## Esophageal functional exploratory tests

Esophageal manometry in patients with BE usually shows a very incompetent LES and also a poor esophageal peristalsis, with low amplitude waves, explaining the presence and persistence of acid reflux in the esophagus [**4**].

Patients with BE have increased gastro-esophageal reflux, and an unusual composition. This type of reflux may evoluate without pain, and due to poor esophageal clearance, the healing of the lesions can be delayed. This is why, patients with BE frequently present with complication of GERD like ulcer, strictures, hemorrhage, but it is less frequent in patients with short segment Barrett’s esophagus. 

## Endoscopic surveillance of Barrett’s esophagus

Taking into consideration the possible development of an adenocarcinoma, the patients with Barrett’s esophagus require endoscopic surveillance after a standardized protocol. Such a protocol was elaborated in 1995 at the 6th World Congress of the International Society for Diseases of the Esophagus and the recommendations were that after histological confirmation of columnar epithelium, patients with BE without dysplasia should undergo an upper endoscopy with biopsy once at every 3 years, patients with low-grade dysplasia, in the first year should undergo upper endoscopy with biopsy at every 6 months, then yearly and patients with a high-grade dysplasia, after second opinion confirmation from a gastro-enterology expert, should undergo esophagectomy.

## Treatment

There is still much controversy in the treatment of patients with Barrett’s esophagus, especially in the presence of dysplasia. The aims of the treatment are gastro-esophageal reflux symptoms control, healing of associated esophagitis and prevention of development of adenocarcinoma. Both medical and surgical treatments are efficient regarding symptoms, but there is lack of confirmation about preventing the development of adenocarcinoma.

In 2005, Yeh and Triadafilopoulos [**5**] proposed an algorithm of surveillance and treatment of the patients with BE, taking into consideration the presence or absence of dysplasia:

1. Patients without dysplasia – PPI treatment for reflux symptoms

2. Patients with histological confirmation of dysplasia and identifiable lesions on endoscopy:

a. EMR with PPI for patients with low-grade dysplasia:

b. Esophagectomy in case of high-grade dysplasia.

3. Patients with histological confirmation of dysplasia but no identifiable lesions:

a. patients with low grade dysplasia: endoscopic surveillance and PPI or endoscopic ablative method (PDT) with PPI;

b. patients with high-grade dysplasia – esophagectomy.

***Medical treatment***

Is represented by antisecretory medication, H2 receptor inhibitory and proton pump inhibitory (PPI), with doses adjusted to symptoms and presence of esophagitis, ulcer, strictures. PPI therapy is very efficient in symptoms control, healing the lesions of esophagitis or ulcer and in prevention of restructuring after endoscopic dilatation. Though, the injury of esophageal mucosa can continue due to alkaline reflux [**6**].

**Fig. 5:**
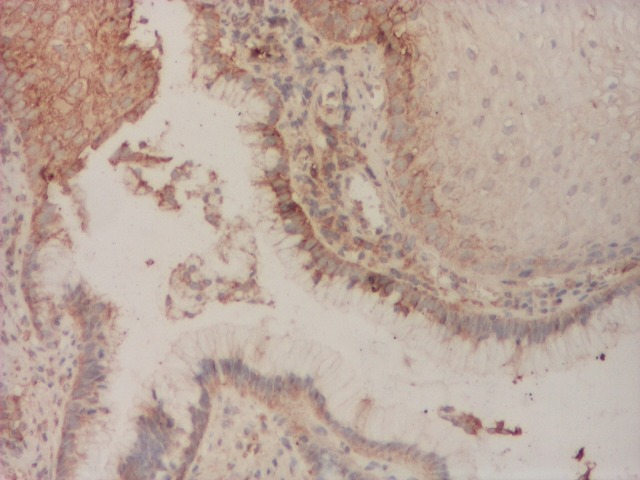
COX2 expression in Barrett’s esophagus, IHC, 40x

Over expression of COX2 (**Fig. 5**) was demonstrated in patients with BE and esophageal adenocarcinoma and recent experimental studies showed that inhibition of COX2 reduces the incidence of esophageal adenocarcinoma through inhibition of angiogenesis and epithelial proliferation as well as the stimulation of apoptosis of the cells with genic mutations. Administration of selective or non-selective COX2 inhibitory, associated with inhibitors of gastric secretion can be a promising method of prevention in the development of adenocarcinoma in patients with BE [**7**].

In patients with good reflux symptoms control on PPI treatment, 24h pH monitoring showed the persistence of acid reflux. Up to 80% of the patients on PPI twice daily experience nocturnal reflux episodes, when esophageal pH drops under 4 for more than 1 hour [**8**]. In some patients, for obtaining almost complete aclorhydria, one must associate to high dose of PPI twice daily, one dose of H2 receptor inhibitory at bedtime [**9**]. Such aggressive therapy remains controversy. The sustainers of such therapy are saying that acid reflux is the main factor which implicates in carcinogenesis and that the complete elimination would prevent the development of cancer [**10**]. Studies on intestinal metaplasia culture cells showed that brief exposure to acid reflux is a stimulus for proliferation [**11**, **12**].

***Surgical treatment in patients with BE***

*The aims of the surgical treatment are*:

1. Controlling the gastro-esophageal reflux symptoms

2. Eliminating acid and alkaline reflux in esophagus 

3. Preventing and stopping the evolution of complications 

4. Preventing the extension of the epithelium with metaplasia 

5. Regression of metaplasia

6. Preventing the evolution towards dysplasia, thus avoiding the development of esophageal adenocarcinoma [**13**].

Surgical therapy addresses especially the mechanical defects of SEI and is curative in 85-93% of the patients.

Nissen fundoplication is the most accepted technique in patients with normal esophageal motility. In patients with poor esophageal peristaltis, one should take into consideration a partial fundoplication (for example: Toupet, Dorr). The main goal of the operation is to reestablish antireflux barrier, without the side effects of medication. Several technique principles are generally accepted: establishing a minimum 12mmHg pressure of the LES, a sufficient abdominal esophageal length, good gastric fornix mobilization, closure of the diaphragmatic defect and avoiding the injury of the vagus nerve [**14**].

Endoscopic surveillance after antireflux procedure showed macroscopic regression of the columnar epithelium in 62% of the patients, while histological regression was present in 40% of the patients [**15**].

Patients with fundoplication require endoscopic surveillance because dysplasia and adenocarcinoma can occur, and the failure of the procedure can be a risk factor for it. 

Antireflux surgery is more effective as treatment for LGD than medical therapy because it is able to control both the acid and biliopancreatic reflux [**16**].

Some studies showed that, after fundoplication, acid reflux persists in almost 35% of patients with Barrett’s esophagus, and at 10 years from the operation, up to 95% of patients present with duodenum-gastro-esophageal reflux. Esophageal ulcer, stricture and esophagitis are present in 15-30% of patients followed long term and in 16% of patients the metaplasia progresses. 6% of patients with Barrett’s esophagus develop low grade dysplasia and 3.4% adenocarcinoma. Regression of low grade dysplasia is found in 45% of the patients [**17**].

Some studies show that surgical treatment, fundoplication, could be more efficient in prevention of deaths related to cancer in patients with BE [**18**].

Some authors say that the progression of Barrett’s esophagus towards adenocarcinoma cannot be substantially prevented by antireflux surgery [**19**].

An alternative therapy with good results is the association of duodenal diversion with PPI treatment. It has 91% clinical success rate and it eliminates almost completely the acid and duodenum-gastro-esophageal reflux. There was no case of progression to dysplasia or adenocarcinoma. In 60% of patients the regression was seen from low grade dysplasia to metaplasia [**20**].

**Fig. 6:**
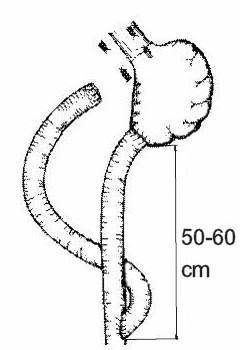
Troncular vagotomy, antrectomy and duodenal diversion with gastro-jejunal Roux anastomosis

**Fig. 7:**
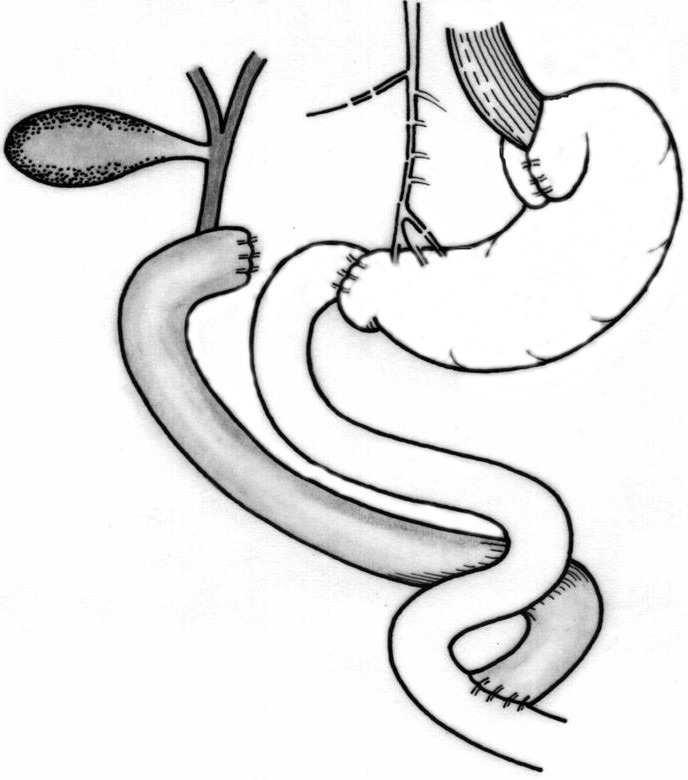
Highly selective vagotomy, Nissen fundoplication and duodenal switch

Troncular bilateral vagotomy associated with antrectomy and total duodenal diversion with gastro-jejunal Roux anastomosis (**Fig. 6**) is indicated especially in complicated stage of the disease, when esophageal motility is severely damaged. For better control of gastro-esophageal reflux, some authors recommend a highly selective vagotomy, fundoplication and duodenal switch (DeMeester) (see **Fig. 7**) [**21**].

***Endoscopic treatment***

In 1993, Berenson demonstrated that the removal of the columnar epithelium is followed by replacement with squamous epithelium in the absence of an acid environment. From this point on, many endoscopic ablative methods were imagined.

Endoscopic therapies conserve esophageal integrity and offer the patients a better quality of life, regarding that patients with BE and HGD have a very low possibility of metastasis. The opponents of endoscopic treatment rely on the identification of areas of adenocarcinoma or HGD on the esophagectomy resection specimen, unidentified preoperatively.

Although HGD and adenocarcinoma in situ can be treated with esophagectomy, the morbidity and mortality, as well as the patients’ associated pathology conducted to increased interest in the endoscopic therapy.

Local endoscopic treatment associated with PPI should be considered in patients with HGD or intramucos adenocarcinoma, who refuse surgical treatment or are not recommended such a treatment because of their age and associated pathology. 

***Photo Dynamic Therapy (PDT)*** implies the endovenous administration of a photosensibilizer substance that concentrates in abnormal esophageal mucosa. The activation of this substance, by applying a red light through the endoscope generates oxygen and other cyto-toxic components that destroy the mucosa. In comparison with other ablative methods, PDT is the only one that can be applied for unidentifiable endoscopic lesions, being indicated in HGD because of the frequent synchronic lesions and adenocarcinoma association.

Ablation on Barrett’s epithelium with HGD usually requires two or more sessions. PDT is efficient in only 50% of the patients [**22**]. The rate of strictures can be up to 30% in patients treated with PDT. Subsquamous intestinal metaplasia without dysplasia can develop in 4,9% of the patients and is also possible the development of adenocarcinoma.

***Multipolar Electro Coagulation (MEC)***

The principle of this method is thermal energy release at the level of abnormal esophageal mucosa, using two or more electrodes located in the tip of the endoscope. The success of the ablation of Barrett’s epithelium decreases to 25% once the length of BE is more that 4cm. The advantages of this method are that it is easily available and cheap. But, sometimes, it requires several sessions of treatment to obtain complete ablation [**23**].

***Laser Therapy***

Lasers generate an intense ray of light which can determine the thermal destruction of abnormal esophageal mucosa if it is pointed towards it. The depth of the destruction depends on the type of laser that is used, varying from 3-4 mm with Nd:YAG to 1 mm with KTP or Argon laser. Subsquamous intestinal metaplasia can develop in 20-90% of these patients [**24**].

***Argon Plasma Coagulation (APC)***

The high frequency monopolar electric current is conducted towards tissue passing through an ionized argon catheter, placed in the working canal of the endoscope. The depth of the lesion is less than the one obtained with PDT and laser. Complications can occur in 24% of the patients: pneumoperitoneum, subcutaneous emphysema, pain, ulceration, bleeding, perforation and even death [**25**]. APC is the only safe and efficient method for the ablation of Barrett’s esophagus after antireflux surgery [**26**].

***Endoscopic Mucosa Resection (EMR)*** is an ablative technique that removes the mucosa along with a part of submucosa. It is indicated in patients with identifiable lesions of HGD or in situ adenocarcinoma on endoscopy. Unlike the other ablative methods, the resection specimens obtained can be evaluated regarding stage, free margins and histology. It is frequently followed by a diffuse therapy like APC or PDT. The use of this method is limited by the presence of lymph node metastasis. The most important predictive factor for lymph node metastasis is the presence of submucosa invasion, seen on endoscopic ultrasonography, this finding being considered a contraindication of the method [**27**].

Radiofrequency ablation uses an esophageal balloon that contains several bipolar electrodes, closely related, that alternate polarity. The electrodes are attached to a radiofrequency generator that can deliberate a variable quantity of energy, which can be selected by the operator. The depth of the ablation is increasing with the quantity of energy applied [**28**].

***Crioablation*** uses liquid Nitrogen at low pressure. The method induces apoptosis and necrosis at very low temperatures (-78 to -158°C). The destruction of Barrett’s epithelium with variable grades of dysplasia is followed by complete reversion to squamous epithelium. The method is relatively new and needs more studies for assessing efficiency and complications.

***Multimodal therapy***

Combining several ablative methods was used for the optimization of the treatment, exploiting the advantages of each method. For example, PDT is frequently followed by laser ablation, MEC or EMR for the residual Barrett’s esophagus. The time will validate if multimodal therapy is the ideal way of implementing ablative therapies [**29**].

***Esophagectomy***

HGD is one of the indications for esophageal resection [**30**]. Early detection of progression towards carcinoma in BE patients, before the obstructive symptoms appear, offers the best chance of cure. Patients with HGD have frequent areas of adenocarcinoma. Some studies showed an incidence of adenocarcinoma of 50-73% on esophagectomy specimens in patients operated for HGD [**31**].

Other arguments for esophagectomy are the facts that HGD is frequent multifocal and esophagectomy removes the entire Barrett mucosa, preventing the development of other areas of dysplasia [**32**].

Taking into consideration the morbidity and mortality associated with esophagectomy, the fact that HGD is progressing towards adenocarcinoma in 19-26% of patients, some authors recommend a selective approach, with intensive endoscopic surveillance and esophagectomy in case of proven adenocarcinoma. In the effort to decrease morbidity and mortality, in medical centers with experience in esophageal surgery, the esophagectomy is made through minimally invasive approach, in order to reduce operative trauma. Minimally invasive esophagectomy is an attractive alternative for patients with HGD, because of lower postoperative mortality and length of stay [**33**, **34**].

Patients with HGD and those who are not fitted for esophagectomy because of advanced age and associated pathology, demonstrated in repeated endoscopic examinations, that they are candidates for endoscopic ablative methods associated with PPI treatment [**35**, **36**].

**Fig. 8:**
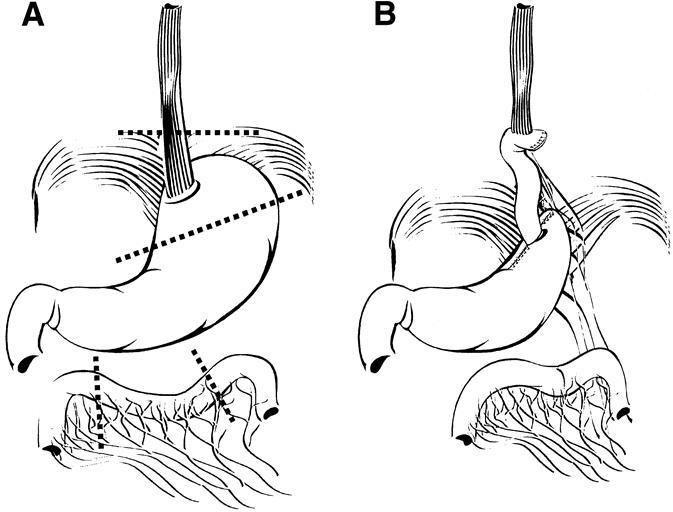
Merendino procedure

**Fig. 9:**
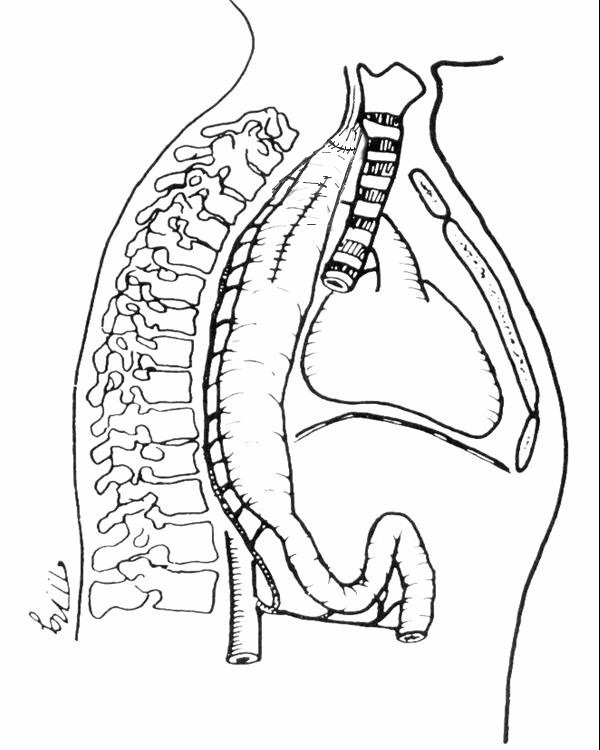
Abdomino-cervical esophagectomy with gastric pull-up

Esophagectomy procedures for patients with HGD are limited esophagectomy with jejunal loop interposition (Merendino – **Fig. 8**) and transhiatal esophagectomy (Orringer – **Fig. 9**).

Endoscopic ablative methods for metaplasia and dysplasia in BE are of limited use, because of the difficulty of establishing indication, risk of complications and the risk of metaplasia and even dysplasia that can develop underneath healed squamous epithelium. The progression towards cancer in patients with BE cannot be substantially prevented through antireflux surgery or PPI, although in some patients, it can be seen the regression of dysplasia, especially after highly selective vagotomy, fundoplication and total duodenal diversion. Still, antireflux surgery is useful for abolishing chronic gastro-esophageal reflux, implicated in progression of metaplasia towards adenocarcinoma. 
